# The association between ambient air pollution and scarlet fever in Qingdao, China, 2014–2018: a quantitative analysis

**DOI:** 10.1186/s12879-021-06674-8

**Published:** 2021-09-21

**Authors:** Fachun Jiang, Tao Wei, Xiaowen Hu, Yalin Han, Jing Jia, Bei Pan, Wei Ni

**Affiliations:** 1 Department of Acute Infectious Diseases, Qingdao Municipal Center for Disease Control and Prevention, Qingdao Institute of Prevention Medicine, Qingdao City, Shandong Province People’s Republic of China; 2 Qingdao Women and Children’s Hospital, Qingdao University, No.6 Tongfu Road, Qingdao City, 266000 Shandong Province People’s Republic of China

**Keywords:** Air pollution, Scarlet fever, Relative risk, Distributed lag non-linear model

## Abstract

**Background:**

We conducted a distributed lag non-linear time series analysis to quantify the association between air pollution and scarlet fever in Qingdao city during 2014–2018.

**Methods:**

A distributed lag non-linear model (DLNM) combined with a generalized additive mixed model (GAMM) was applied to quantify the distributed lag effects of air pollutions on scarlet fever, with daily incidence of scarlet fever as the dependent variable and air pollutions as the independent variable adjusted for potential confounders.

**Results:**

A total of 6316 cases of scarlet fever were notified, and there were 376 days occurring air pollution during the study period. Scarlet fever was significantly associated with air pollutions at a lag of 7 days with different relative risk (RR) of air pollution degrees [1.172, 95% confidence interval (CI): 1.038–1.323 in mild air pollution; 1.374, 95% CI 1.078–1.749 in moderate air pollution; 1.610, 95% CI 1.163–2.314 in severe air pollution; 1.887, 95% CI 1.163–3.061 in most severe air pollution].

**Conclusions:**

Our findings show that air pollution is positively associated with scarlet fever in Qingdao, and the risk of scarlet fever could be increased along with the degrees of air pollution. It contributes to developing strategies to prevent and reduce health impact from scarlet fever and other non-vaccine-preventable respiratory infectious diseases in air polluted areas.

**Supplementary Information:**

The online version contains supplementary material available at 10.1186/s12879-021-06674-8.

## Background

Scarlet fever is an infectious disease caused by toxin-producing strains of the bacteria Streptococcus pyogenes (Group A Streptococcus, GAS), which occurs most commonly in association with pharyngitis [1, 2]. Although the effective antibiotics, hygiene and nutrition have been improved in the past decade, the re-emergency of scarlet fever was noted in some areas over the globe, such as South Korea, Vietnam England, UK as well as China [3–9]. Notably, among the areas, China is facing an increasing threat of scarlet fever after implementation of two-child policy from 2011, which had a significant increase in the reports of scarlet fever. Yet, the reason for this increase has been not clear, which is suggested with potential association between microbial, host, meteorological and environmental factors [10]. Due to the rapid economic development and urbanization in China, the frequency and severity of air pollution episodes increased over the last two decades, resulting in a risk of health impacts on an unprecedented scale [11–16]. The emerging cases of scarlet fever and worsening air pollution may suggest a potential linkage, however, few evidence revealed this association in a large population study.

In eastern China, more air pollution events were observed compared with other areas [17]. Qingdao, as an important economic center and a seaport in eastern China, has suffered from air pollution frequently, which is presented as a region with high PM_2.5_ and PM_10_ mass concentrations [18]. What happening with worsening air pollution at the same time is the increasing incidence of scarlet fever, which is a significant threat to the growing child population in Qingdao [19]. Therefore, Qingdao, presented as the site, is appropriate to explore the association between air pollution and scarlet fever. At present, we conducted a distributed lag non-linear time series analysis to quantify the association between them in a large population study in Qingdao, China during 2014–2018, aiming at providing facility to developing strategies for preventing and reducing health impact from scarlet fever and other non-vaccine-preventable respiratory infectious diseases in air polluted areas.

## Methods

### Study site

**Fig. 1:**
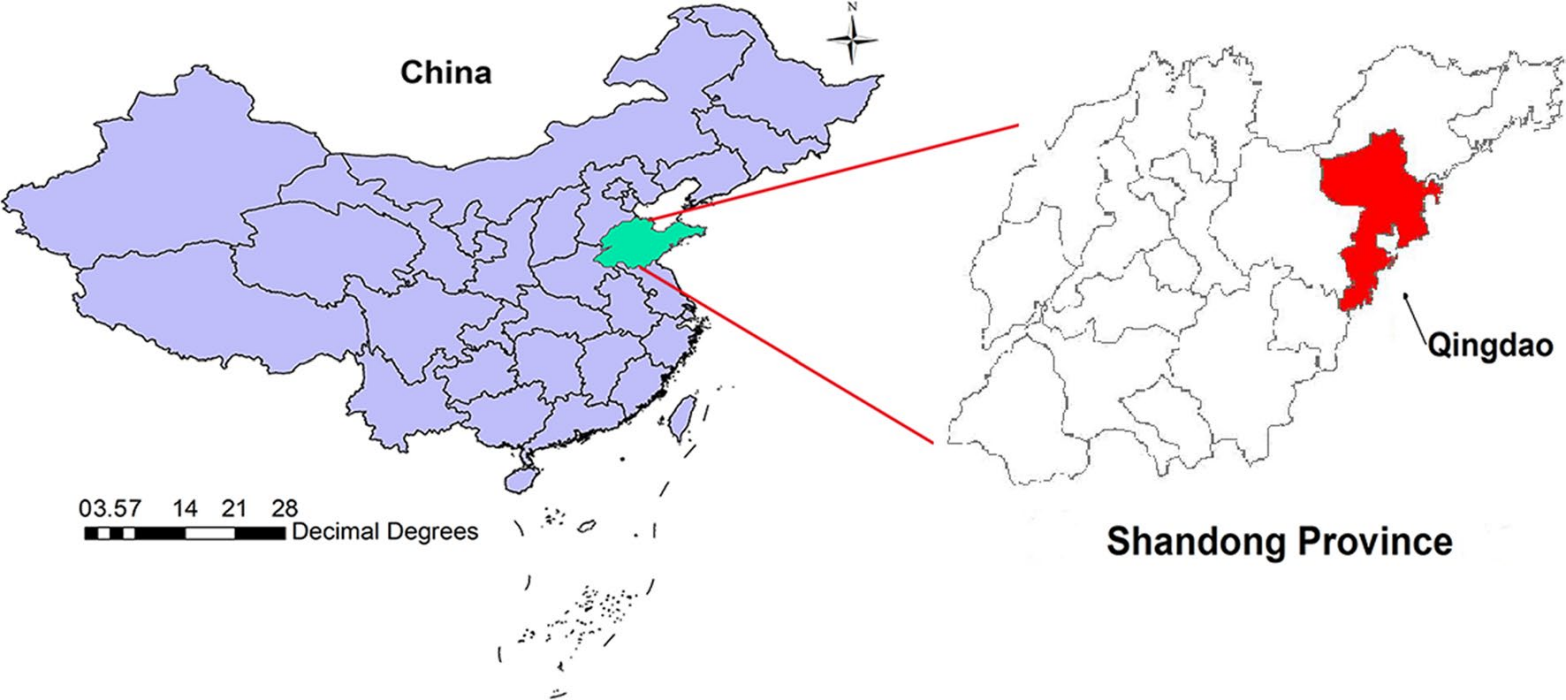
Location of Qingdao in Shandong Province, China (the image depicted in Fig. 1 is our own)

As shown in Fig. 1, Qingdao is a coastal city of Shandong province, which is situated in eastern China between longitude 119°30′–121°00′ E and latitude 35°35′–37°09′ N. The city has a mid-temperate continental monsoon climate with an annual average of 12.7 °C and annual cumulative precipitation of 662.1 mm. Additionally, as a harbor city, Qingdao is the economic center of Shandong province with a population density of 801 persons per km^2^ (in 2014: population = 9,046,200; land size = 11,282 km^2^).

### Data collection and management

#### Data on disease

Resource and collection of disease data was depicted in the previous article written by Rao et al. [20]. Daily data on scarlet fever from 2014 to 2018 in Qingdao were obtained from the Notifiable Disease Surveillance System (NDSS). According to the Chinese Infectious Diseases Law, clinicians must report to the NDSS when they identify any probable, clinical, or laboratory-confirmed case of scarlet fever within 24 h of diagnosis, and it ensures that the morbidity of scarlet fever is a relative real figure of the city. Additionally, all cases of scarlet fever were diagnosed according to the diagnostic criteria for scarlet fever issued by the Ministry of Health of the People’s Republic of China in 2008 [21]. This study was approved by the Ethics Commission of Qingdao Municipal Center for Disease Control and Prevention (Date: 18 Oct, 2019; Number: QFELL-KY-2019-67).

#### Air pollution data

Resource and collection of air pollution data was depicted in the previous article [22]. Air pollution data during 2014–2018 in Qingdao were obtained from China National Environmental Monitoring Center, including data of daily air quality index and air pollutant concentrations, such as PM_2.5_, PM_10_, sulfur dioxide (SO_2_), carbon monoxide (CO), nitrogen dioxide (NO_2_) and ozone (O_3_). According to Ambient Air Quality Standards issued by Ministry of Ecology and Environment of the People’s Republic of China in December 2012, the standard limits of particulate matter with a diameter less 2.5 microns (PM_2.5_), particulate matter with a diameter less 10 microns (PM_10_), SO_2_, CO and NO_2_ concentrations, equivalently to the 24-h means, are 75 μg/m^3^, 150 μg/m^3^, 150 μg/m^3^, 4 mg/m^3^ and 80 μg/m^3^, respectively, followed by the O_3_ concentration limit with 200 μg/m^3^ on eight hours average [22].

The previous article introduced the definition of air pollution and its assessment process in detail, which was also applied in this study [22]. Air pollution is defined as the phenomenon or event that the content of any substance in atmospheric are varied harmfully for ecological stability and the condition of human survival, causing hazards for human, animals, vegetation or material [22]. Severity of air pollution is indicated by different air quality index (AQI) value ranges. AQI is a number used by government agencies to communicate to the public how polluted the air is currently, which is summarized by considering several main air pollutants and calculated by Individual Air Quality Index (IAQI) of each pollutant [22]. IAQI represents the state of individual contaminant. The IAQI was calculated as follows according to the Technical Regulation on Ambient Air Quality Index (on trial):

$$IAQI_{P} = \frac{{IAQI_{Hi} - IAQI_{Lo} }}{{BP_{Hi} - BP_{Lo} }}\left( {C_{P} - BP_{Lo} } \right) + IAQI_{Lo}$$

IAQI_P_ represents the Individual Air Quality Index of P contaminant. C_p_ represents the mass concentration of P contaminant. BP_Hi_ and BP_Lo_ represent the highest and lowest value of concentration limit like C_P_, respectively. IAQI_Hi_ and IAQI_Lo_ represent the Individual Air Quality Index of BP_Hi_ and BP_Lo_, respectively [22].

The AQI was calculated as followed:

$$AQI = \max \left\{ {{\text{IAQI}}_{{1}} ,{\text{IAQI}}_{{2}} {\text{,IAQI}}_{{3}} {,}...{\text{,IAQI}}_{{\text{n}}} } \right\}$$

IAQI represents the Individual Air Quality Index of contaminants. n represents the specific contaminant.

AQI values are divided into four ranges, and each range is assigned a descriptor for air pollution level. According to the Technical Regulation on Ambient Air Quality Index (on trial), air pollution is divided into 4 levels on the basis of AQI value, including mild pollution (AQI: 101–150), moderate pollution (AQI: 151–200), severe pollution (AQI: 201–300) and most severe pollution (AQI: > 300) [22].

#### Meteorological data

Meteorological data from 2014 to 2018 were collected from the China Meteorological Data Sharing Service System (http://cdc.cma.gov.cn/), which includes daily data such as daily cumulative precipitation, daily mean temperature and daily mean air pressure.

### Statistical analysis

First, the distribution of scarlet fever morbidity and air pollution variables were described during the study period. Second, a distributed lag non-linear model (DLNM) combined with a generalized additive mixed model (GAMM) was applied to quantify the distributed lag effects of air pollutions on scarlet fever, with daily morbidity of scarlet fever as the dependent variable and air pollutions as the independent variable adjusted for potential confounders. A quasi-Poisson regression was used to deal with the over dispersion of Poisson distribution. In order to control the potential confounds, factors including meteorological factors, long-term and seasonal trend, day of the week and public holidays were introduced into the model simultaneously. The model is as follows:

$$\begin{aligned} \log \left[ {E(Y_{t} )} \right] & = \alpha + \sum\limits_{p = 0}^{7} {\beta_{p} \left( {Air{\kern 1pt} {\kern 1pt} {\kern 1pt} Pollution_{t - p} ,DF} \right)} + \sum\limits_{q = 0}^{7} {\beta_{q} \left( {Air{\kern 1pt} {\kern 1pt} {\kern 1pt} {\text{Pollutant}}_{t - q} ,DF} \right)} \\ & + NS_{1} \left( {{\text{Prec,}}\,DF} \right) + NS_{2} \left( {Temp,DF} \right) + NS_{3} \left( {{\text{Pressure,}}\,DF} \right) \\ & + NS_{4} \left( {Time,DF} \right) + DOW_{t} + Holiday \\ \end{aligned}$$

Y_t_ denoted the daily morbidity of scarlet fever on day t. α was the intercept. Air Pollution was a categorical variable including non-air pollution, mild air pollution, moderate air pollution, severe air pollution or most severe air pollution, represented by 0, 1, 2, 3 and 4, respectively. The β_p_ was the effect estimate of the air pollution p days before the day of illness. Air Pollutant was a metric variable presenting the concentration of air pollutant. The β_q_ was the effect estimate of a 10-unit of increase of air pollutant concentration (with reference to its standard limit) q days before the day of illness. The NS_1_ (Prec, DF), NS_2_ (Temp, DF), NS_3_ (Pressure, DF) and NS_4_ (Time, DF) were natural cubic splines of daily cumulative precipitation, daily mean temperature, daily mean air pressure and time [as the number of days (1, 2, 3… 1086)], respectively, which were designed to control the effects of meteorological factors, long-term trend and seasonality. DF was the degree of freedom. DOW_t_ was the day of the week on day t, which was a categorical variable (1, 2, 3… 7). Holiday was a binary variable including public holiday or workday, represented by 1 and 0.

All degrees of freedom of variables were selected according to the empirical researches. In order to completely capture the effects of air pollution and air pollutant concentrations on daily morbidity of scarlet fever, the DLNM was applied for air pollution and air pollutants in our study with both 3 degrees of freedom (DF) [23–25]. Using a natural cubic spline, we chose DF as 7 per year for Time to remove long term trends and seasonality impact [25]. Additionally, we used smooth function of natural cubic splines with 3 DF in the model for daily cumulative precipitation, daily mean temperature and daily mean air pressure [26, 27]. Previous studies have shown that the lagged effect of air pollutants on respiratory diseases were usually short [28, 29]. The incubation period of scarlet fever is usually between 1 and 3 days [30]. However, considering the delayed environmental transport of pathogens and delayed onset of clinical symptoms, morbidity of scarlet fever was expected to peak several days after the exposure of air pollution. Therefore, a lag effect at a maximum of 7 days was applied in the DLNM.

Air pollutants usually have a highly interaction effect, which may result in collinearity in the model. In order to avoid the collinearity, the pairwise correlation was applied by spearman correlation analysis in all air pollutants. As shown in Additional file 1: Table S1, there were two pairs with no significant correlation among the six air pollutants, including PM_2.5_-O_3_ (r = − 0.025, P < 0.05) and PM_10_-O_3_ (r = 0.006, P < 0.05). Previous studies found the strong association between PM_2.5_, O_3_ and respiratory infectious disease [31, 32], thus, our study focused on PM_2.5_ and O_3_ as the pollutant variables included in the model to assess their impact.

In order to detect the potential autoregressive correlation of the model, the Durbin–Watson (D–W) test was conducted, and results showed that D–W statistic was 1.84 with the P value of 0.16, revealing that the model with no autoregressive correlation.

Sensitive analysis was performed by altering DF (6–9 per year) for Time, and DF (2–5) for daily cumulative precipitation, daily mean temperature and daily air pressure. R software (version 3.2.2, R Development Core Team 2015) was used to perform all statistical analyses. The “dlnm” package was used to create the DLNM model. All statistical tests were two-sided, and P values with less than 0.05 were considered statistically significant.

## Results

### Description of disease and air pollution

A total of 6316 cases of scarlet fever were notified in the study area over no air pollution and air pollution periods from 2014 to 2018. Descriptive statistics of the scarlet fever morbidity, air pollution and meteorological factors were presented in Table 1, which were significantly different between non-air pollution and air pollution periods. During the study period, there were 376 days occurring air pollution, including 278 days with mild air pollution, 58 days with moderate air pollution, 37 days with severe air pollution and 3 days with most severe air pollution.

**Table 1:** Description of scarlet fever incidence, air pollution and meteorological factors from 2014 to 2018 in Qingdao city

Variables	Period	Mean ± SD	Min	P_25_	Median	P_75_	Max
Daily morbidity of scarlet fever(1 × 10^8^)	No air pollution period	3.7 ± 3.7	0	1.1	2.2	5.4	23.9
	Air pollution period*	4.3 ± 4.2	0	1.1	3.3	6.5	22.6
PM_2.5_ (μg/m^3^)	No air pollution period	32.6 ± 16.9	0	19.8	29.0	43.0	95.0
	Air pollution period*	89.0 ± 44.5	0	58.0	83.5	107.0	304.0
PM_10_ (μg/m^3^)	No air pollution period	70.0 ± 66.0	0	48.0	66.0	90.0	165.0
	Air pollution period*	156.0 ± 63.1	0	119.0	150.0	186.0	455.0
SO_2_ (μg/m^3^)	No air pollution period	17.9 ± 12.4	2.0	9.0	15.5	23.0	79.0
	Air pollution period*	33.8 ± 22.2	2.0	18.0	28.0	43.0	132.0
CO (mg/m^3^)	No air pollution period	0.7 ± 0.6	0.2	0.5	0.6	0.8	8.6
	Air pollution period*	1.3 ± 0.8	0.4	0.9	1.1	1.5	12.6
NO_2_ (μg/m^3^)	No air pollution period	31.9 ± 14.4	3.0	21.0	30.0	40.0	107.0
	Air pollution period*	48.7 ± 18.9	9.0	34.0	46.0	62.8	111.0
O_3_ (μg/m^3^)	No air pollution period	94.5 ± 32.2	0	68.0	95.0	119.0	161.0
	Air pollution period*	113.0 ± 60.7	0	58.3	99.0	168.8	254.0
Cumulative precipitation (mm)	No air pollution period	1.9 ± 7.7	0	0	0	0	121.4
	Air pollution period*	0.3 ± 2.6	0	0	0	0	39.2
Average temperature (°C)	No air pollution period	14.3 ± 9.6	− 12.9	6.4	15.7	22.6	30.5
	Air pollution period*	11.9 ± 9.4	− 4.4	3.7	9.9	20.1	30.6
Average air pressure (KPa)	No air pollution period	1000.8 ± 9.2	988.3	1000.4	1008.4	1015.8	1032.3
	Air pollution period*	1009.3 ± 8.7	987.8	1001.9	1010.4	1016.0	1027.6

*SD* standard deviation, *Min* minimum, *P25* the 25th percentile, *P75* the 75th percentile, *Max* maximum

*P < 0.05 vs. non-flooded month

### Association between air pollution and scarlet fever

**Fig. 2:**
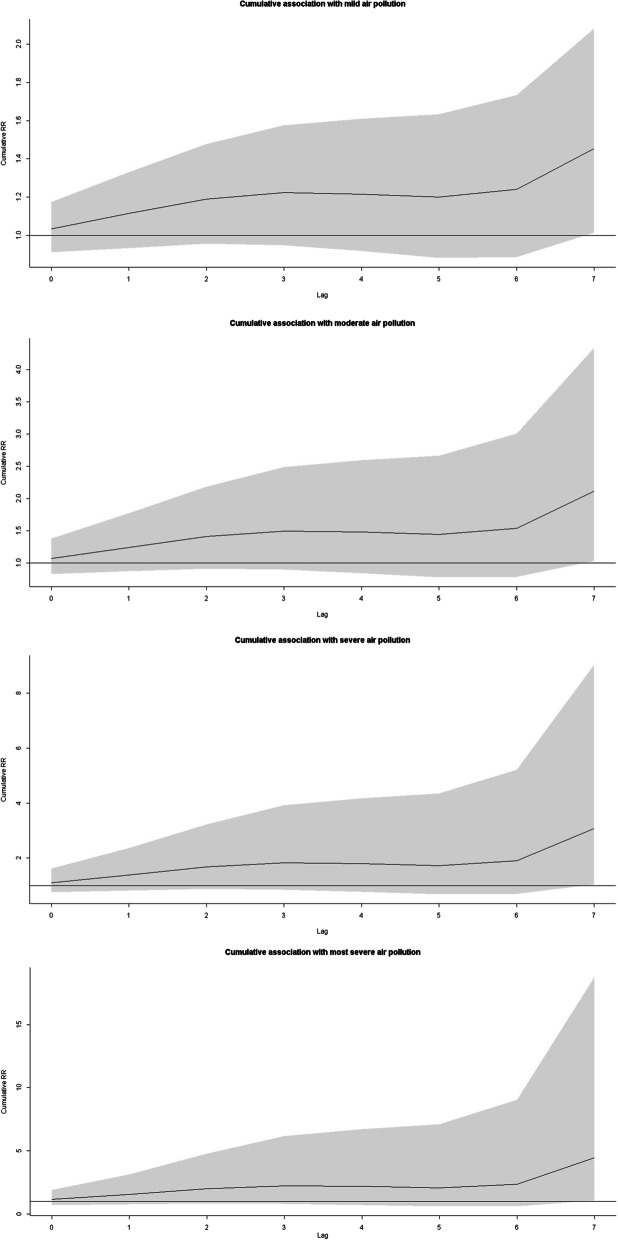
The cumulative relative risks of different degrees of air pollution at a lag of 0–7 days (including mild pollution, moderate pollution, severe pollution and most severe pollution)

After controlling for daily cumulative precipitation, daily mean temperature, daily mean air pressure, seasonality, long-term trends, DOW and public holidays, results from the DLNM showed that the morbidity of scarlet fever was significantly associated with air pollutions, and the lag effects were presented in Table 2. The relative risks (RRs) of air pollution on scarlet fever were only significant at a lag of 7 days, which were 1.172 (95% CI 1.038–1.323) in mild air pollution, 1.374 (95% CI 1.078–1.749) in moderate air pollution, 1.610 (95% CI 1.163–2.314) in severe air pollution and 1.887 (95% CI 1.163–3.061) in most severe air pollution. Moreover, the cumulative effects of air pollutions on scarlet fever were presented in Fig. 2, and the cumulative RRs at a lag of 0–7 days were 1.454 (95% CI 1.015–2.082) in mild air pollution, 2.114 (95% CI 1.031–4.334) in moderate air pollution, 3.073 (95% CI 1.046–9.023) in severe air pollution and 4.467 (95% CI 1.062–18.785) in most air pollution.

**Table 2:** The RRs of air pollution on the risk of scarlet fever from the DLNM model

Lags	Mild	Moderate	Severe	Most severe
Lag0	1.034 (0.912–1.174)	1.071 (0.831–1.379)	1.108 (0.758–1.619)	1.147 (0.691–1.902)
Lag1	1.077 (0.994–1.168)	1.161 (0.987–1.363)	1.251 (0.983–1.592)	1.348 (0.977–1.859)
Lag2	1.066 (0.980–1.159)	1.137 (0.961–1.344)	1.212 (0.943–1.558)	1.292 (0.924–1.806)
Lag3	1.029 (0.957–1.106)	1.058 (0.916–1.224)	1.089 (0.877–1.353)	1.121 (0.839–1.497)
Lag4	0.994 (0.925–1.068)	0.988 (0.856–1.141)	0.982 (0.792–1.219)	0.977 (0.732–1.303)
Lag5	0.987 (0.908–1.073)	0.974 (0.825–1.151)	0.962 (0.749–1.235)	0.949 (0.680–1.325)
Lag6	1.034 (0.954–1.120)	1.069 (0.910–1.254)	1.105 (0.869–1.405)	1.142 (0.829–1.573)
Lag7	1.172 (1.038–1.323)*	1.374 (1.078–1.749)*	1.610 (1.163–2.314)*	1.887 (1.163–3.061)*

*Mild* mild air pollution, *Moderate* moderate air pollution, *Severe* severe air pollution, *Most severe* most severe air pollution

*P < 0.05

**Fig. 3:**
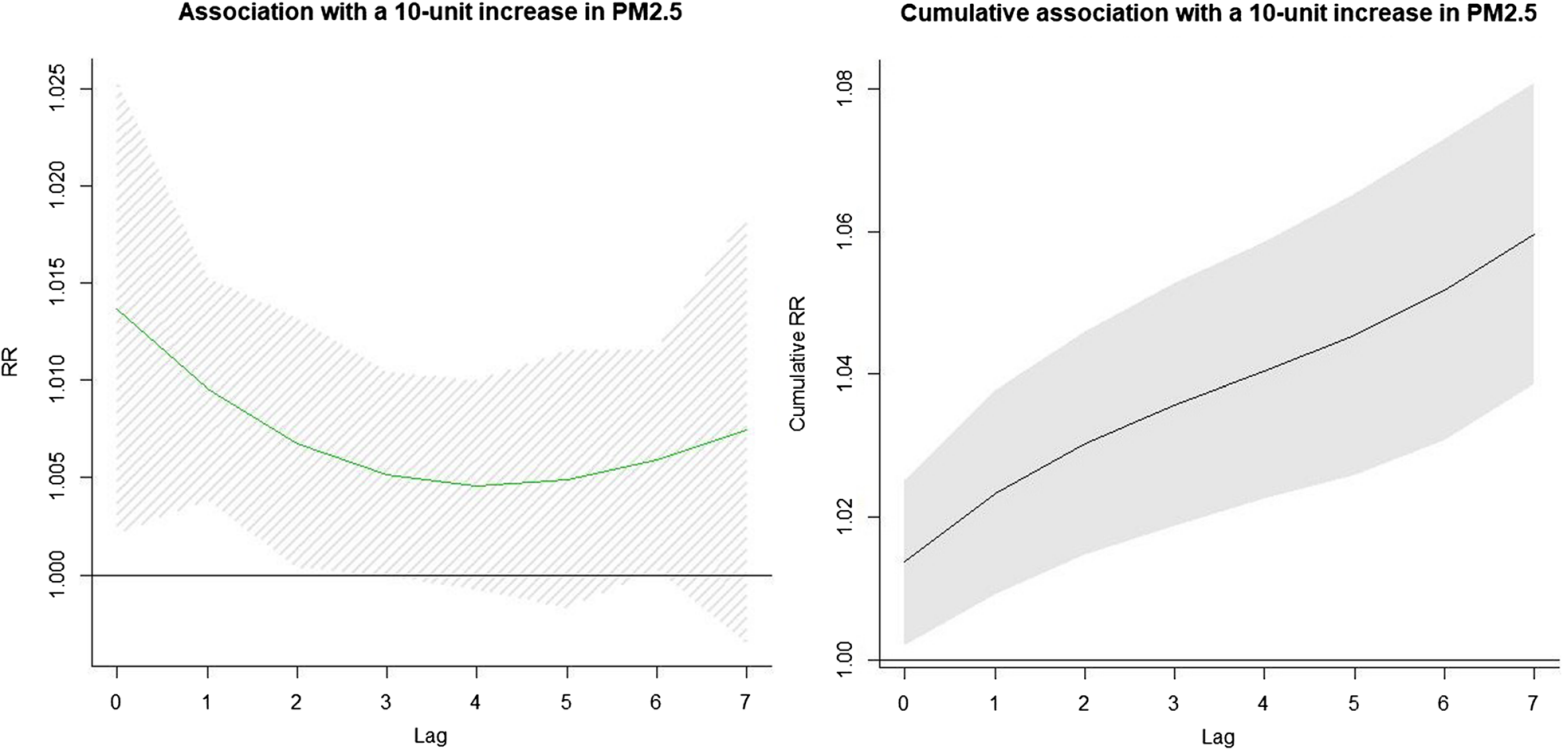
The relative risks and cumulative relative risks of a 10-unit increase of PM_2.5_ concentration at a lag of 0–7 days (with reference to standard limit of 75 μg/m^3^)

**Fig. 4:**
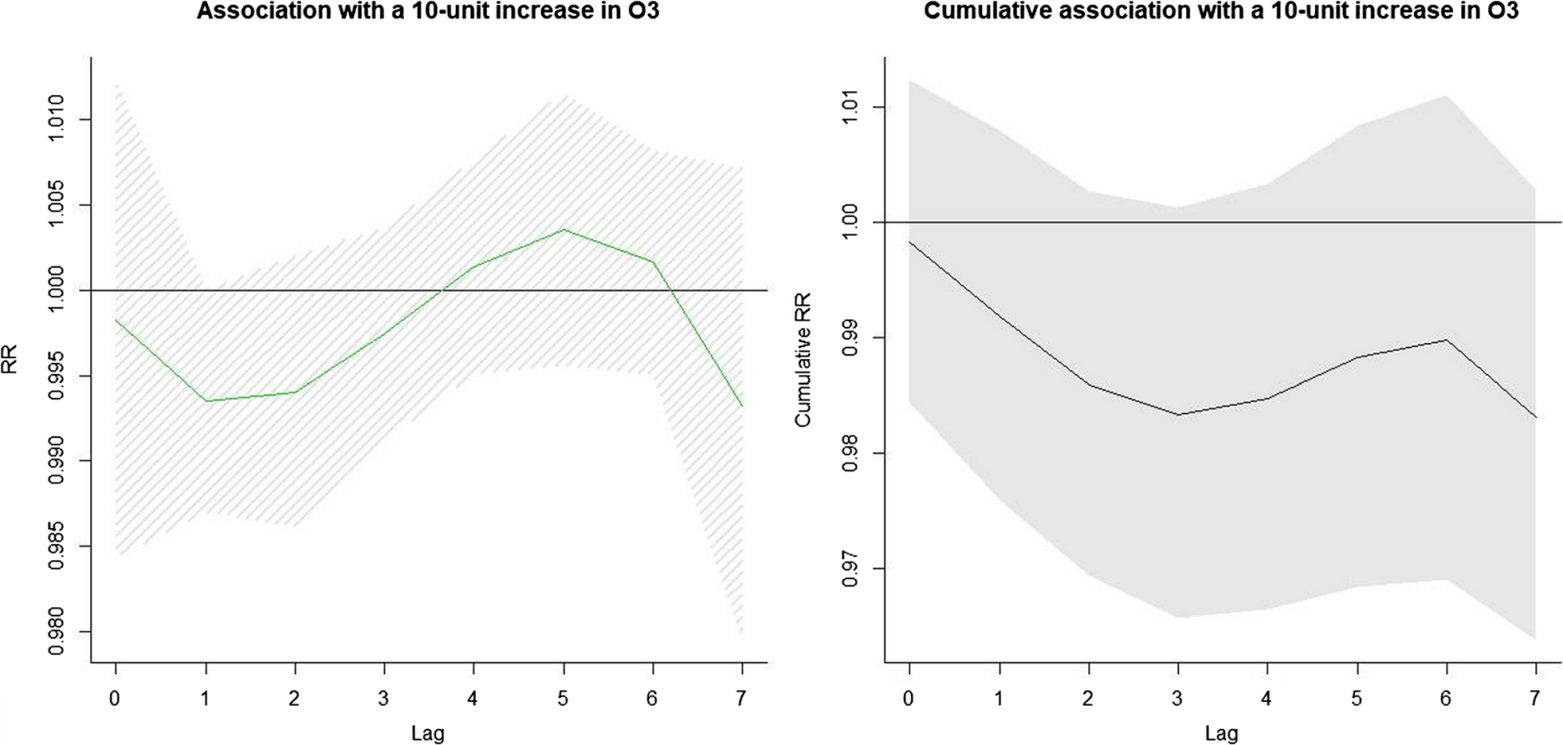
The relative risks and cumulative relative risks of a 10-unit increase of O_3_ concentration at a lag of 0–7 days (with reference to standard limit of 200 μg/m^3^)

As shown in Fig. 3, the association between a 10-unit of increase of PM_2.5_ concentration and the morbidity of scarlet fever was significantly detected from the model (with reference to 75 μg/m^3^). The RRs were significant at a lag of 0–2 and 6 days, with its maximum at a lag of 0 day (1.014, 95% CI 1.003–1.025), and the cumulative RR at lag 0–7 days was 1.060 (95% CI 1.039–1.081). However, there was no significant association detected between a 10-unit increase of O_3_ concentration and morbidity of scarlet fever (with reference to 200 μg/m^3^), which was showed in Fig. 4.

### Sensitivity analyses

Sensitivity analysis was conducted to check whether our coefficient estimates were robust. The effects changed little when changing DF (2–5) for daily cumulative precipitation, daily mean temperature, and daily mean air pressure, and we found that the effects estimated at a lag of 7 days did not change substantially (Additional file 1: Figure S1). Similar effects of air pollutions on scarlet fever were observed when changing DF (6–9 per year) for time (Additional file 1: Figure S2).

## Discussion

Previous studies reveal that scarlet fever is related to meteorological factors [33, 34], however, the potential risk environmental factors have been considered to be more. Our results from DLNM suggested the lagged and cumulative effect of air pollutions on scarlet fever alongside controlling for the potential impact of meteorological factors, day of the week, holiday, seasonality and long-term trend. A viewpoint supported by Liu et al. is also appropriate for our research [35], which is that although this study is based on Qingdao city only, the real impact of scarlet fever due to air pollution might be much greater, given the large population at risk and frequent air pollutions in China. Results from this study might be applicable to most cities in coastal areas of north China, because air quality and climates in those places were similar with that in Qingdao.

Air pollution is the fifth leading global risk factor for public health, which contributes substantially to disease burden [36, 37]. Due to the implementation of policies and plans to reduce the adverse effects of air pollution on public health in China, the air quality at most regions has been improving since 2013 and achieved the decrease of national annual mean concentrations of air pollutants between 2004 and 2018 [13]. However, air pollution remains severe, and its subsequent health effects still persist. In Qingdao, there were 376 days occurring air pollution between 2014 and 2018, accounting for one fifth of this period. During air pollution days, the mean concentrations of pollutants were significantly higher than non-air pollution days, which increased along with air pollution levels.

To our knowledge, it has been the first time that a study evaluated the risk of air pollution on scarlet fever based on air pollution levels not only pollutant concentrations. Previous studies mainly focused on the air pollutant concentrations to evaluate the association with diseases [27, 30, 33]. Although using the concentration could appropriately present the impact of air pollutants, it just indicates a single pollutant, which seems to be far from adequate for assessing the impact of air pollutions. Air pollution is a complex environmental problem, and it integrates the statuses of various pollutants. Therefore, it should consider integration of various air pollutants at certain moment to analyze the association with diseases. AQI is considered as a summary assessment of ambient air pollutants, aiming at expressing the concentrations of main pollutants on a common scale where effects human health. According to the AQI, air pollution is classified into four levels, including mild pollution, moderate pollution, severe pollution and most severe pollution. Compared with concentrations of air pollutants, the degrees of air pollution might be more interesting in estimating the association between air pollution and scarlet fever, which could present the overall situation of air quality.

In our study, results of the DLNM showed that air pollutions were associated with increased risks of scarlet fever at a lag of 0–7 days. Moreover, it suggested that the risk of air pollutions on scarlet fever could increase along with air pollution levels. Compared with good air quality, the worse air quality may increase the risk of scarlet fever. This could be significant for local government to make advance policies for protecting population health when air pollution is occurring. Additionally, due to the explosion of the child population under the two-child policy in China, the non-vaccine-preventable childhood disease such as scarlet fever might be a potential risk [10, 38], which further increases the exposure population for possible risk of scarlet fever associated with air pollutions. In the recent years of China, the awareness of air pollution and its health implications have been increased significantly, and a series of corresponding measures have been implemented including substantial investments in the improvement of air quality and a multidimensional control strategy aiming at reduce emissions from vehicles and fuels [39–41]. All these actions are very important to decrease the threat of air pollutions merging scarlet fever and other non-vaccine-preventable respiratory infectious diseases in China.

We suppose that the impact of air pollutions on scarlet fever is most depended on the effects of air pollutants. Our results revealed a positive association between daily mean concentration of air pollutant and scarlet fever morbidity, and the cumulative RR of a 10-unit increase of PM_2.5_ concentration at a lag 0–7 days was significantly evaluated (1.060, 95% CI 1.039–1.081). However, there was no significant association detected between O_3_ concentration and scarlet fever. The risk estimate for PM_2.5_ found in our study was consistent with earlier findings in Beijing [42]. Previous studies tried to investigate the mechanisms of the damage effects of PM_2.5_, but the biological mechanisms underlying the association between air pollutants and scarlet fever remain elusive. One of the reasons maybe such exposures to elevated concentrations of PM_2.5_ over short periods may irritate airways in the human respiratory system and potentially increase susceptibility to respiratory infections [43]. Studies suggested that there were three pathways which may promote this possible situation, including injury from free radical peroxidation, imbalanced intracellular calcium homeostasis and inflammatory injury [43]. However, future patient-level and mechanistic research should be done to prove the findings.

Compared with other studies analyzing the association between air pollution and scarlet fever, a significant feature of our study is that we use daily data to detect this association. Different from weekly and monthly data, daily data is more accurate to assess the impact of air pollutions. Appling weekly or monthly data have to face a fact that this would underestimate the effect of extreme pollution events by averaging its impact on a long temporal scale. Using daily data to analyze the association could avoid this situation, and it could identify the degrees of air pollution for more accurate assessment of the impact of air pollution.

Limitations of our study should be acknowledged. Firstly, due to lack of case data, we just evaluated the effect of air pollution on overall population, and cannot analyze the effects among different gender and age groups. Secondly, the effects of many factors, such as population, available health services and hygiene, social and economic status, were not included in this analysis due to unavailable data. Thirdly, we did not analyze the effect of air pollutions on scarlet fever cases by GAS types. In addition, under-reporting is an inevitable issue, which could lead to an underestimation of the impact of air pollutions on scarlet fever.

## Conclusion

In conclusion, air pollution is positively associated with scarlet fever in Qingdao, and the risk of scarlet fever is increased along with the degrees of air pollution. Our findings contribute to developing local strategies to prevent and reduce health impact from scarlet fever and other non-vaccine-preventable respiratory infectious diseases in air polluted areas.

## Supplementary Information

**Additional file 1: Results of spearman correlation and sensitivity analysis.**

## Data Availability

The datasets used during the study are available from the corresponding authors on reasonable request.

## Abbreviations

GAS: Group a streptococcus
GAMM: Generalized additive mixed model
DLNM: Distributed lag non-linear model
RR: Relative risk
CI: Confidence interval
NDSS: Notifiable disease surveillance system
AQI: Air quality index
IAQI: Individual air quality index
PM_2.5_: Particulate matter with a diameter less 2.5 microns
PM_10_: Particulate matter with a diameter less 10 microns
SO_2_: Sulfur dioxide
CO: Carbon monoxide
NO_2_: Nitrogen dioxide
O_3_: Ozone
DF: Degree of freedom
