# Supplementary material for: The association between ambient air pollution and scarlet fever in Qingdao, China, 2014–2018: a quantitative analysis
Source: BMC Infect Dis. 2021 Sep 21;21:987. doi: 10.1186/s12879-021-06674-8 (PMC8456591; doi:10.1186/s12879-021-06674-8)
Supplement: Supplementary file 1 — Additional file 1: Results of spearman correlation and sensitivity analysis. [file 12879_2021_6674_MOESM1_ESM.pptx]

## Slide 1
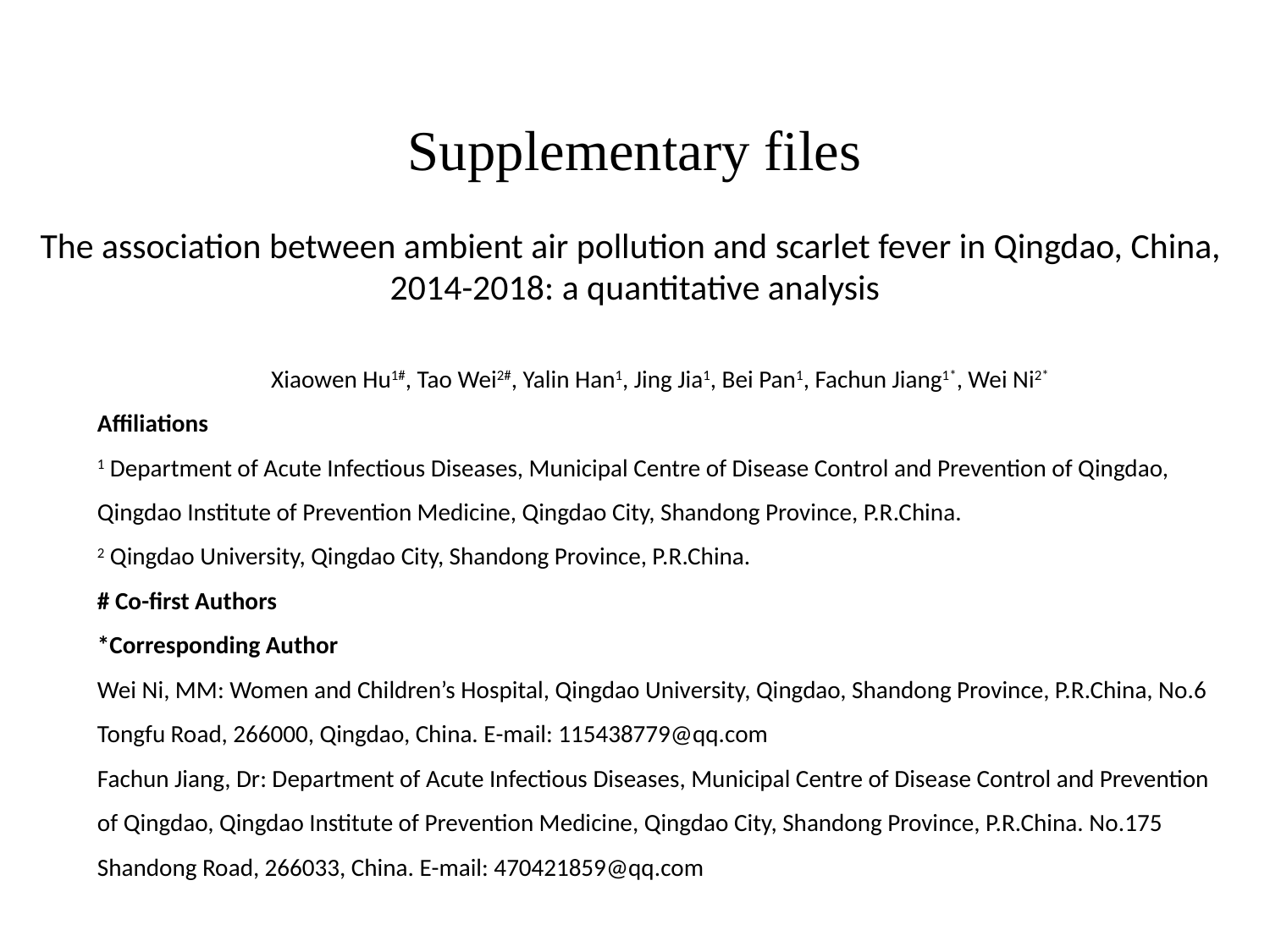

Supplementary files
The association between ambient air pollution and scarlet fever in Qingdao, China,
2014-2018: a quantitative analysis
Xiaowen Hu1#, Tao Wei2#, Yalin Han1, Jing Jia1, Bei Pan1, Fachun Jiang1*, Wei Ni2*
Affiliations
1 Department of Acute Infectious Diseases, Municipal Centre of Disease Control and Prevention of Qingdao, Qingdao Institute of Prevention Medicine, Qingdao City, Shandong Province, P.R.China.
2 Qingdao University, Qingdao City, Shandong Province, P.R.China.
# Co-first Authors
*Corresponding Author
Wei Ni, MM: Women and Children’s Hospital, Qingdao University, Qingdao, Shandong Province, P.R.China, No.6 Tongfu Road, 266000, Qingdao, China. E-mail: 115438779@qq.com
Fachun Jiang, Dr: Department of Acute Infectious Diseases, Municipal Centre of Disease Control and Prevention of Qingdao, Qingdao Institute of Prevention Medicine, Qingdao City, Shandong Province, P.R.China. No.175 Shandong Road, 266033, China. E-mail: 470421859@qq.com

## Slide 2
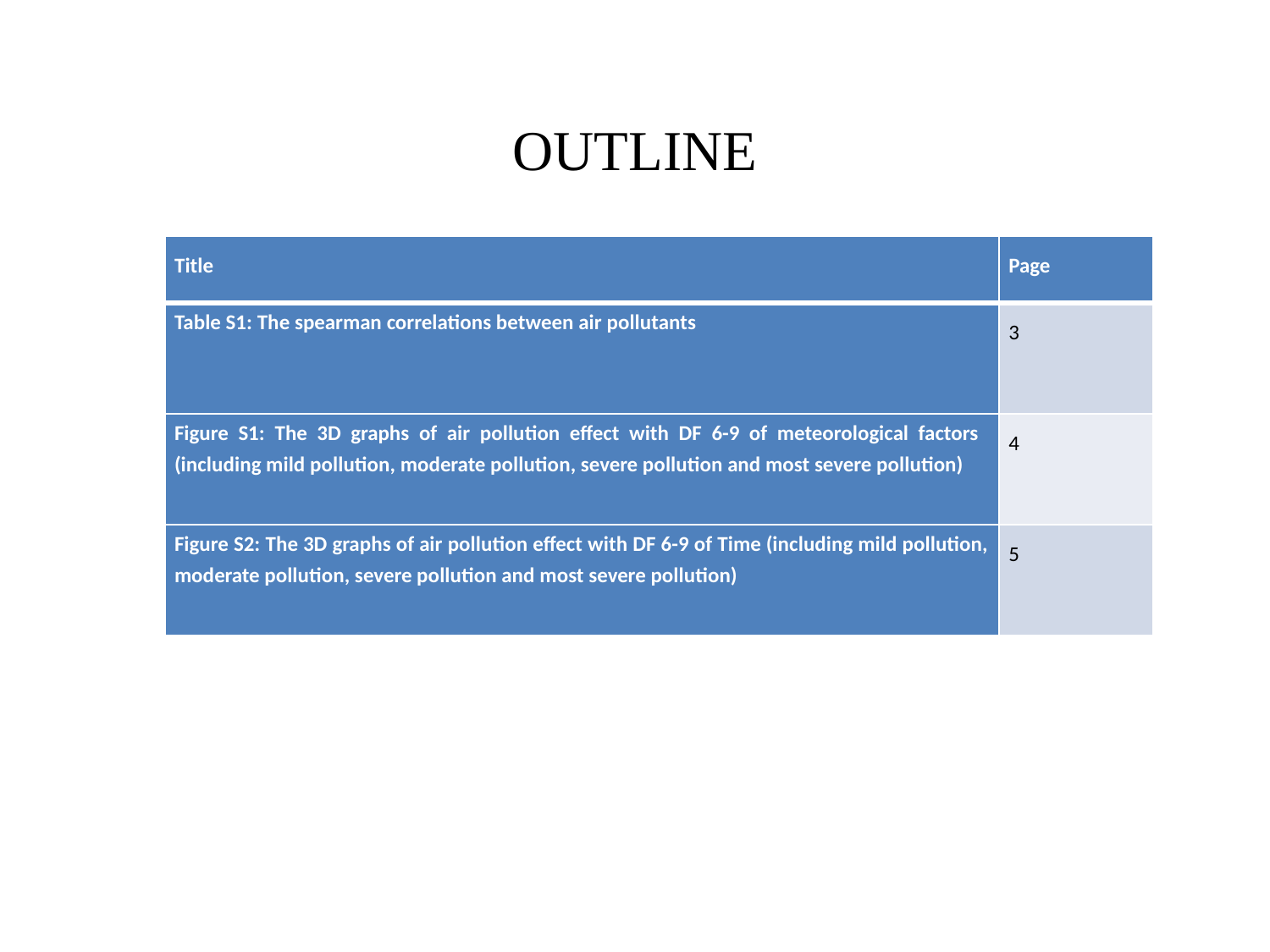

OUTLINE
| Title | Page |
| --- | --- |
| Table S1: The spearman correlations between air pollutants | 3 |
| Figure S1: The 3D graphs of air pollution effect with DF 6-9 of meteorological factors (including mild pollution, moderate pollution, severe pollution and most severe pollution) | 4 |
| Figure S2: The 3D graphs of air pollution effect with DF 6-9 of Time (including mild pollution, moderate pollution, severe pollution and most severe pollution) | 5 |

## Slide 3
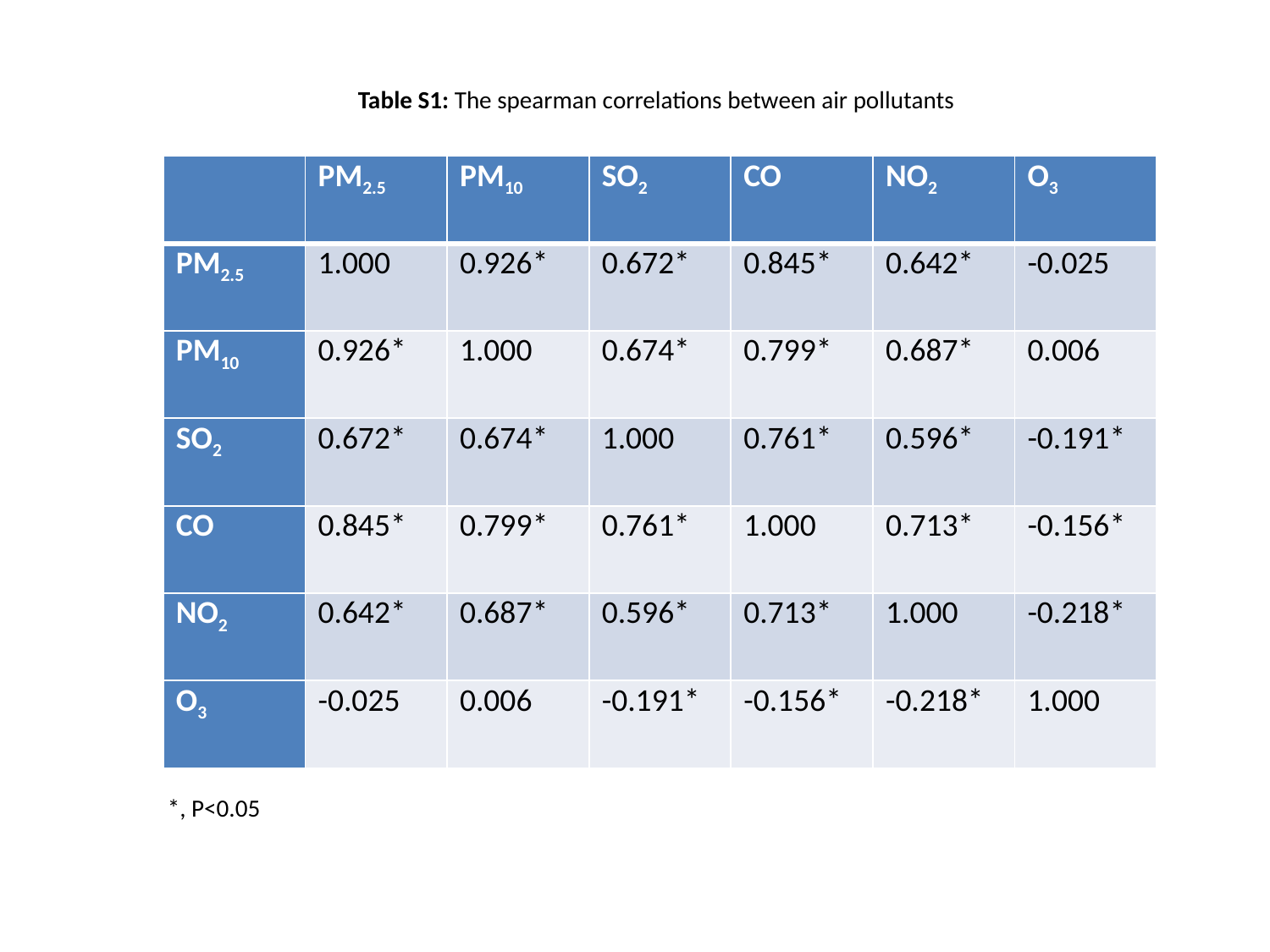

Table S1: The spearman correlations between air pollutants
| | PM2.5 | PM10 | SO2 | CO | NO2 | O3 |
| --- | --- | --- | --- | --- | --- | --- |
| PM2.5 | 1.000 | 0.926\* | 0.672\* | 0.845\* | 0.642\* | -0.025 |
| PM10 | 0.926\* | 1.000 | 0.674\* | 0.799\* | 0.687\* | 0.006 |
| SO2 | 0.672\* | 0.674\* | 1.000 | 0.761\* | 0.596\* | -0.191\* |
| CO | 0.845\* | 0.799\* | 0.761\* | 1.000 | 0.713\* | -0.156\* |
| NO2 | 0.642\* | 0.687\* | 0.596\* | 0.713\* | 1.000 | -0.218\* |
| O3 | -0.025 | 0.006 | -0.191\* | -0.156\* | -0.218\* | 1.000 |
*, P<0.05

## Slide 4
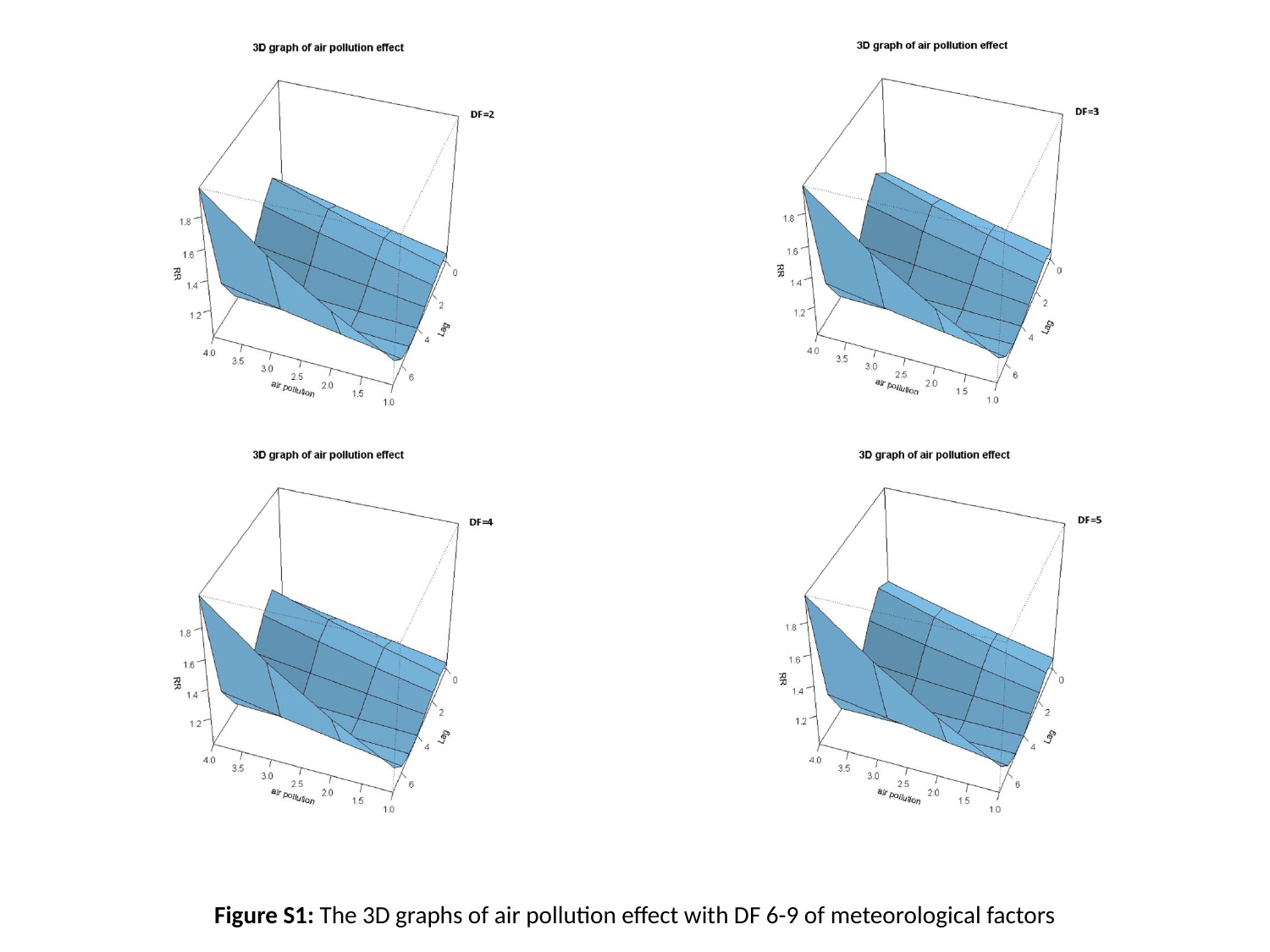

Figure S1: The 3D graphs of air pollution effect with DF 6-9 of meteorological factors

## Slide 5
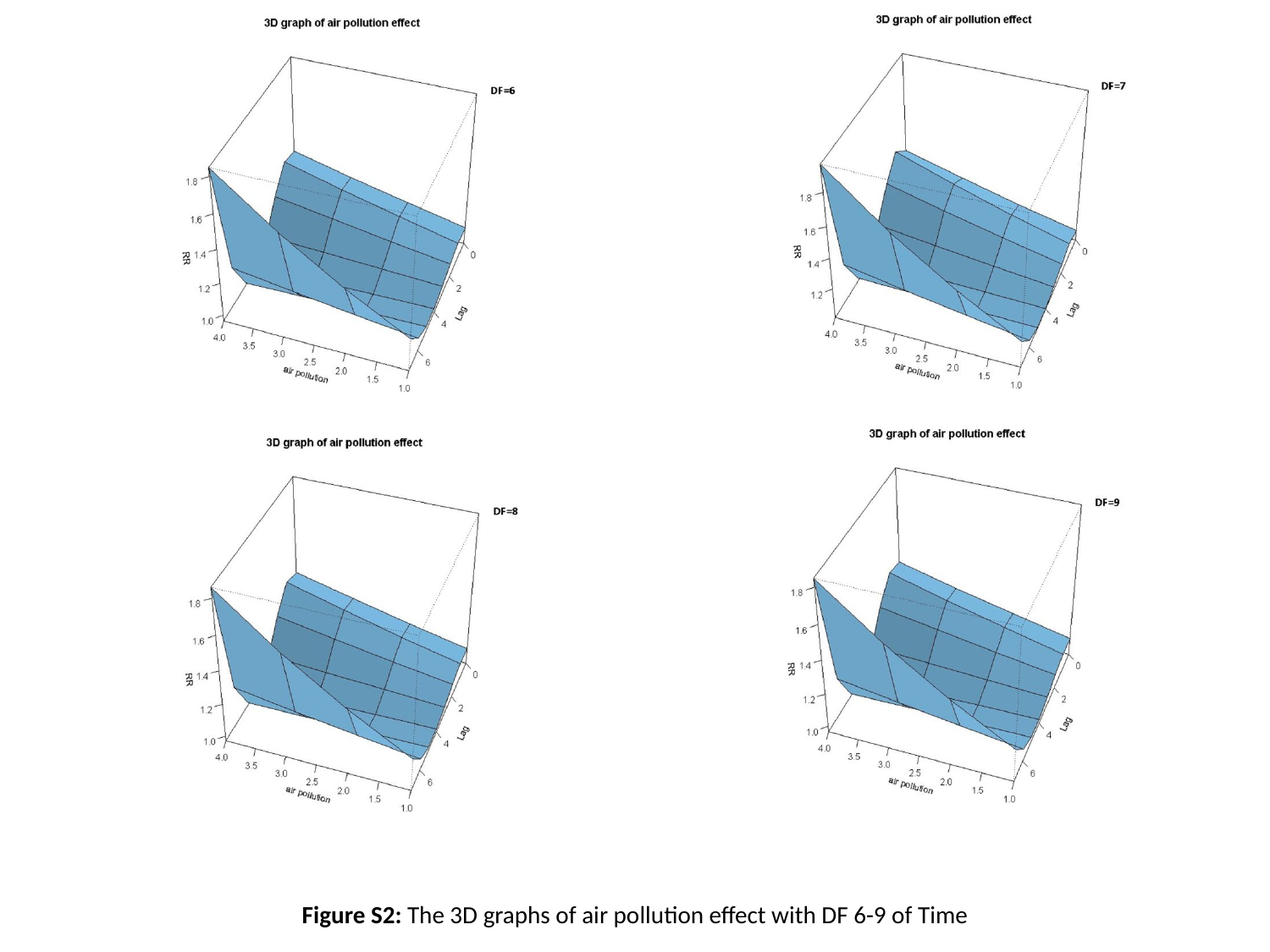

Figure S2: The 3D graphs of air pollution effect with DF 6-9 of Time
